# Supplementary material for: Cascading events during the 1650 tsunamigenic eruption of Kolumbo volcano
Source: Nat Commun. 2023 Oct 26;14:6606. doi: 10.1038/s41467-023-42261-y (PMC10603052; doi:10.1038/s41467-023-42261-y)
Supplement: Supplementary file 1 — Supplementary Information [file 41467_2023_42261_MOESM1_ESM.pdf]

Supplement

Sensitivity analysis

To test the effects of simulation parameters and the interaction between wave fields produced by the slope failure and the explosion, we performed 44 numerical simulation runs with VolcFlow. The simulations sim 01 to sim 20 were dedicated to parameter tests for the slope failure. We used the slide plane defined in Supplement S1 and tested density values of 1,250 kg/m³, 1,500 kg/m³, 1,750 kg/m³, and 2,000 kg/m³ and yield strengths of 5 kPa, 7.5 kPa, 10 kPa, 20 kPa, and 50 kPa, resulting in 20 parameter combinations (**Fig. S1**). The simulations showed a general trend toward higher tsunami waves for greater densities and smaller yield strengths, which is expected as these control the kinetic energy of the sliding mass that is transferred into the water column. It should be noted that density values of 1,750 kg/m³ and 2,000 kg/m³ are too high, according to the analysis of pumice samples obtained from Kolumbo's cone[1], which indicated a bulk deposit density between 1,250 and 1,500 kg/m³. All simulations using a density of 1,250 kg/m³ resulted in too small a wave field (**Fig. S1**). Likewise, simulations using a density of 1,500 kg/m³ and yield strengths of 20 kPa and 50 kPa resulted in too small a wave field (**Fig. S1**). The simulations assuming a density of 1,500 and yield strengths of 5 kPa, 7.5 kPa, 10 kPa, and 20 kPa were the best-suited simulations. In the absence of direct constraints of the slide kinematics, we adopted a density of 1,500 kg/m³ and a yield strength of 7.5 kPa for further simulations combining the slope failure with an explosive eruption (described below).

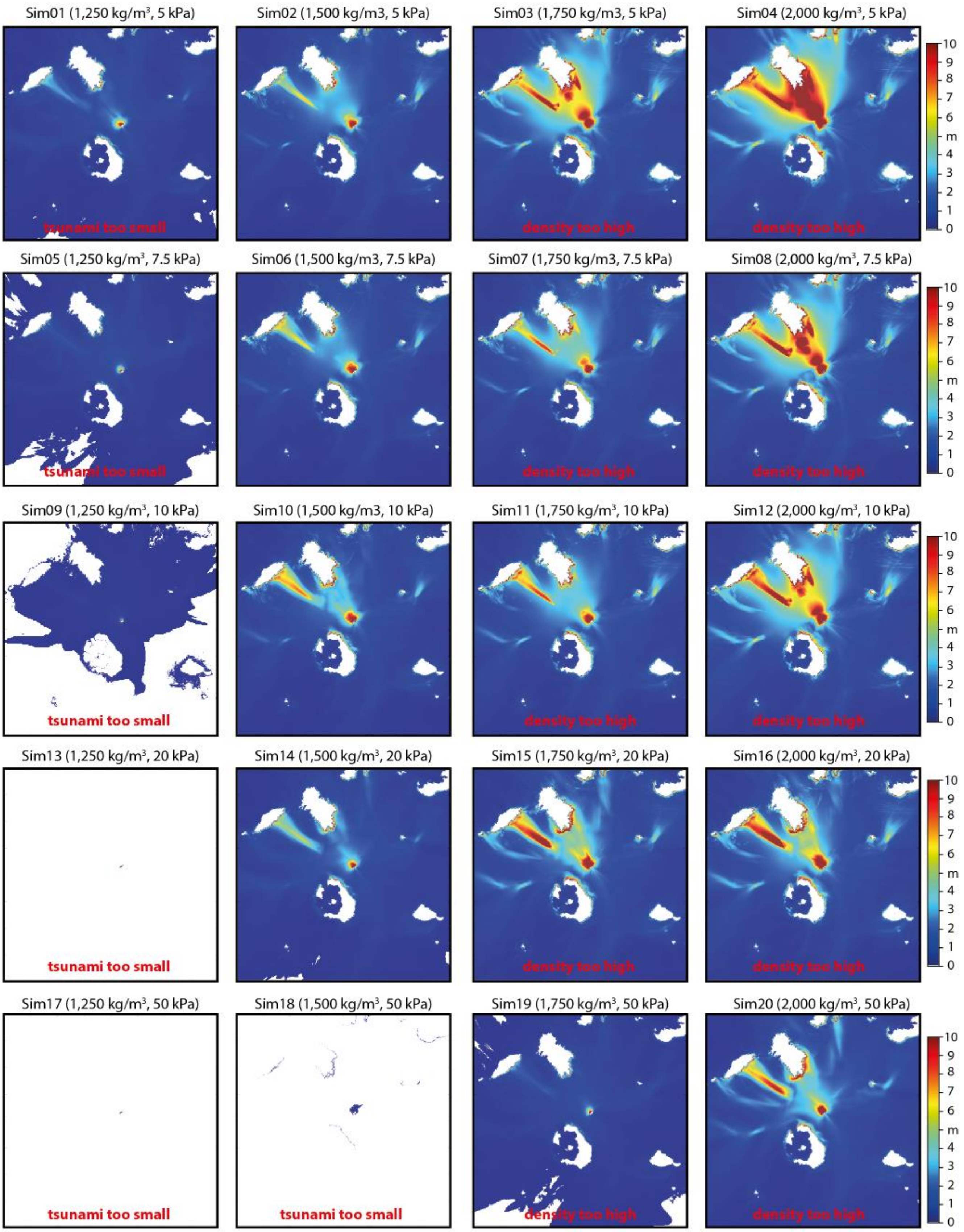

Figure S1 | Maximum tsunami heights for simulations of a slope failure (sim 001 to sim 020). Parameter combinations tested are density (in kg/m3) and yield strength (in kPa).

We performed four simulations for the tsunami genesis by an explosive eruption, following the approach by Ulvrova et al.[2], which requires an estimation of the peak wave height. We simulated peak wave heights of 100 m, 125 m, 150 m, and 240 m. The simulations demonstrate how greater peak wave heights resulted in higher tsunami wave fields, as expected (**Fig. S2**).

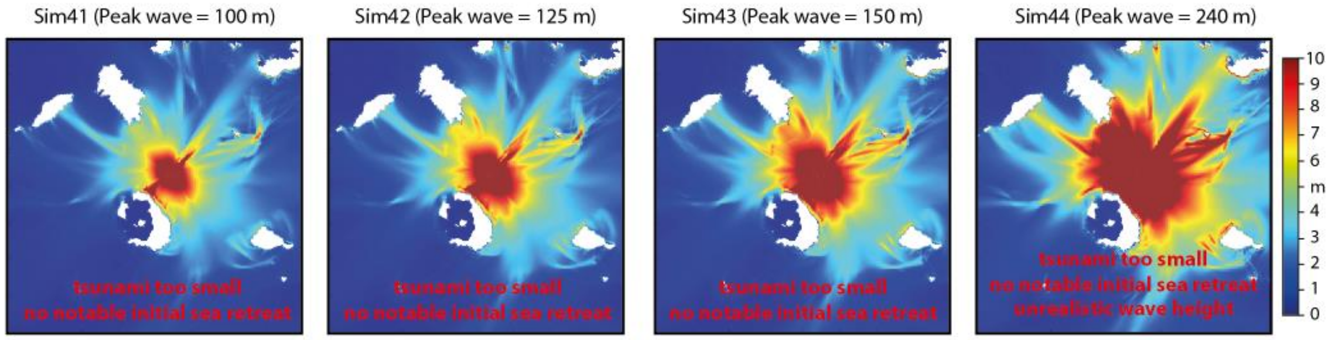

Figure S2 | Maximum tsunami heights for simulations of an explosive eruption (sim 041 to sim 044).

We then performed 20 additional simulations of a combined slope failure and explosion source mechanism. As mentioned earlier, we adopted a best estimate for density (1,500 kg/m^3) and yield strength (7.5 kPa) for the slope failure, and combined this scenario with an explosion with peak wave heights of 100 m, 125 m, 150 m, and 240 m (**Fig. S3**). As the wave fields from both source mechanisms will superimpose themselves, the time delay between slope failure and the explosion is important, but unknown. Therefore, we simulated a broad range of 1 to 5 minute time delays, resulting in 20 parameter combinations. Since the average crater rim depth is 135 m, the simulations assuming a peak wave height of 240 m have to be considered unrealistic. As expected, the time delay between slope failure and explosion is critical in determining whether an initial sea retreat (caused by the slope failure induced tsunami) at Kamari and Perissa is observed or not. Simulations with 1 and 2 minute time delays show no notable initial sea retreat at these two key locations (e.g. in sim 021 in **Fig. S15**). To validate the results, we defined threshold tsunami heights that should be reached to be consistent with historic observations. The precise locations of these historic observations are unknown, and the locations of the tsunami tide gauges do not always represent the location of maximum wave height for a given region (this location varies slightly from simulation to simulation). Therefore, we defined threshold tsunami heights that are smaller than the maximum observed wave heights: 7.5 m at northern Santorini, 5 m at Perissa and Kamari, and 10 m at southern Ios and Sikinos. Following these criteria, and the required initial sea retreat at Perissa and Kamari, only simulations sim 024, sim 028, sim032, sim 038, and sim 039 fulfill all requirements. This highlights the importance of the time delay between different interacting tsunami source mechanisms. **Figs. S5 – S26** show virtual tide gauges and the maximum tsunami heights for all 44 simulations carried out in our sensitivity testing.

Supplementary references

1. Cantner, K., Carey, S., & Nomikou, P. Integrated volcanologic and petrologic analysis of the 1650 AD eruption of Kolumbo submarine volcano, Greece. Journal of Volcanology and Geothermal Research, 269, 28-43 (2014).
2. Ulvrova, M., Paris, R., Nomikou, P., Kelfoun, K., Leibrandt, S., Tappin, D. R., & McCoy, F. W. (2016). Source of the tsunami generated by the 1650 AD eruption of Kolumbo submarine volcano (Aegean Sea, Greece). Journal of Volcanology and Geothermal Research, 321, 125-139 (2016).

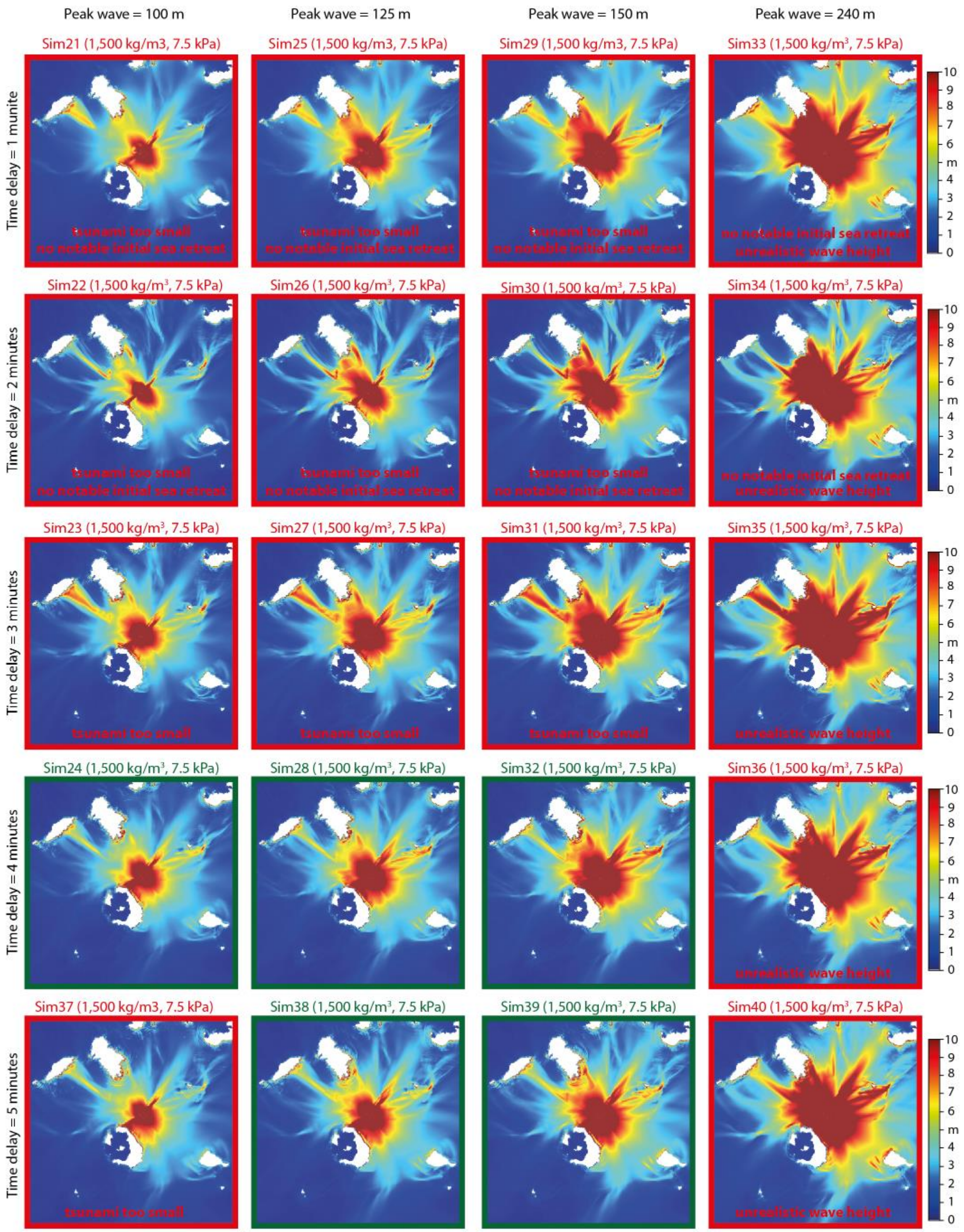

Figure S3 | Maximum tsunami heights for simulations of a combination of slope failure and an explosive eruption (sim 021 to sim 040). Different simulations have different combinations of time delay (from initiation of slope failure to explosion) and peak wave height (from the explosion). All

combinations assume our best estimate of slide density and yield strength (1500 kg/m3 and 7.5 kPa, respectively). Red boxes denote inconsistency with historical accounts. Green boxes denote consistency with all historical accounts.

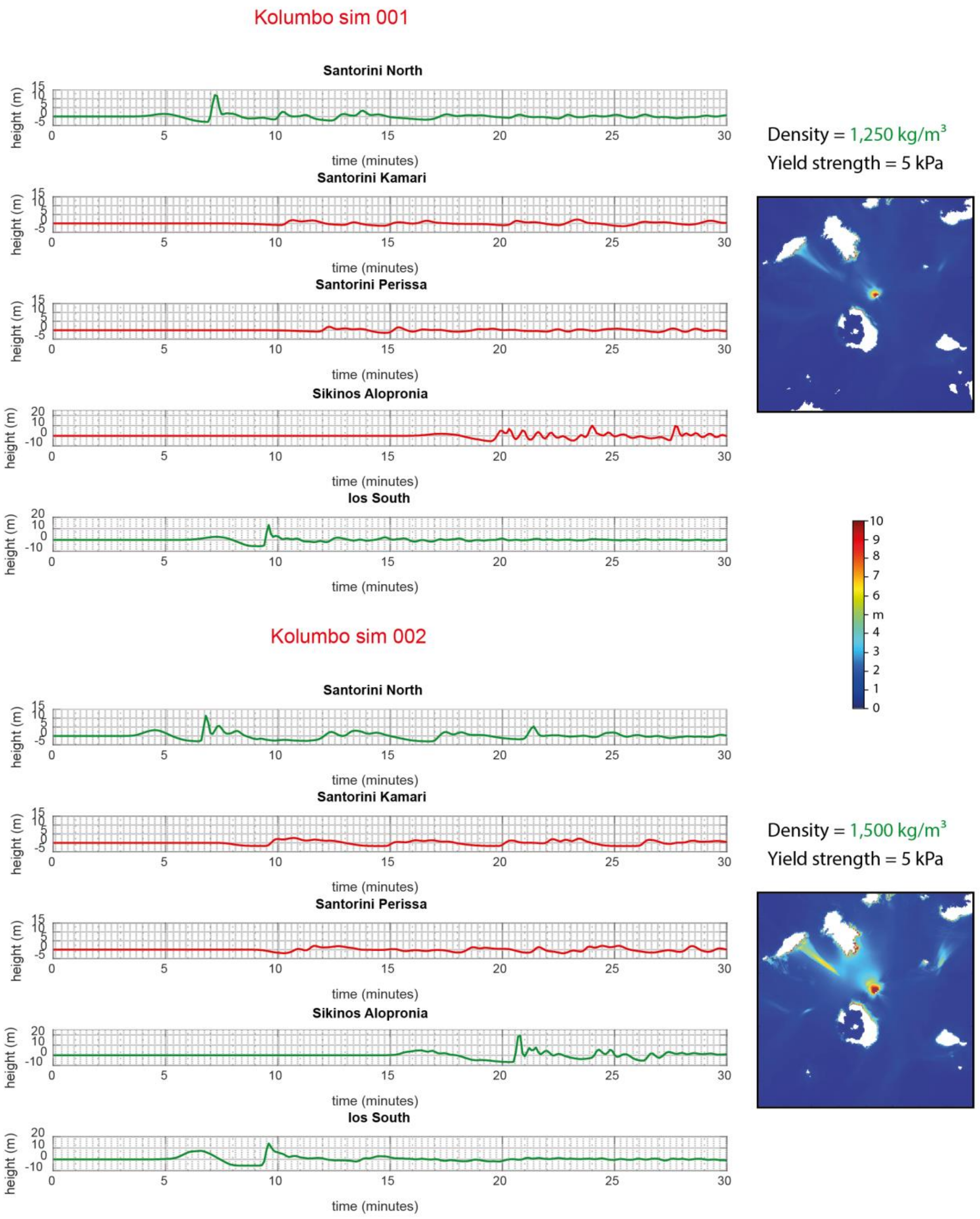

Figure S4 | Virtual tide gauges for simulations sim 001 and sim 002 (red: inconsistent with historical accounts; green: in agreement with historical accounts). Threshold tsunami heights from eyewitness accounts used to evaluate the simulation results: 7.5 m at northern Santorini, 5 m at Perissa, 5 m Kamari, 10 m at southern Ios and 10 m at Sikinos. A direct transfer of inundation observations to tsunami height for Sikinos is not possible and thus the Sikinos threshold is less reliable compared to the direct tsunami height information available for the other locations.

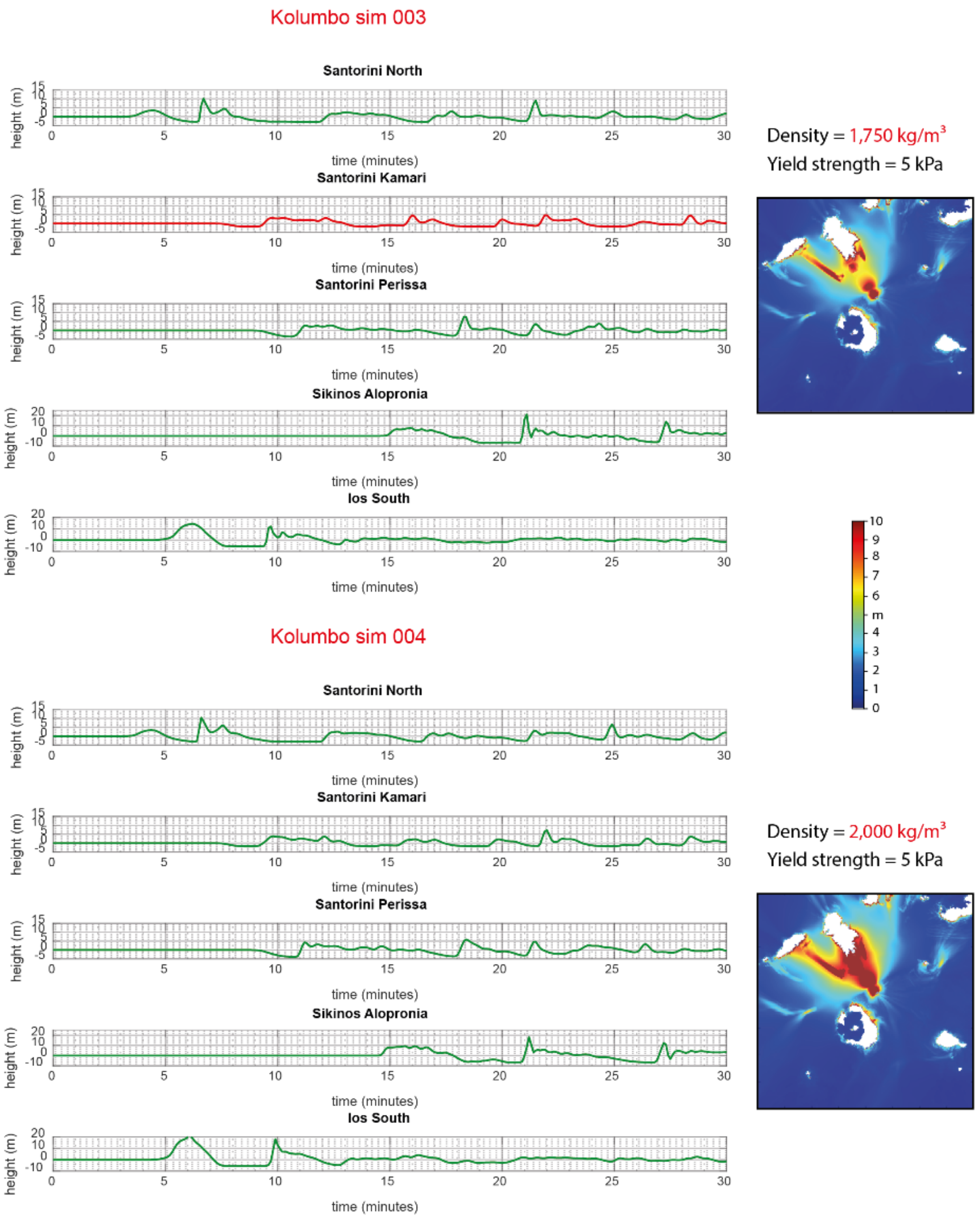

Figure S5 | Virtual tide gauges for simulations sim 003 and sim 004 (red: inconsistent with historical accounts; green: in agreement with historical accounts). Threshold tsunami heights from eyewitness accounts used to evaluate the simulation results: 7.5 m at northern Santorini, 5 m at Perissa, 5 m Kamari, 10 m at southern Ios and 10 m at Sikinos. A direct transfer of inundation observations to tsunami height for Sikinos is not possible and thus the Sikinos threshold is less reliable compared to the direct tsunami height information available for the other locations.

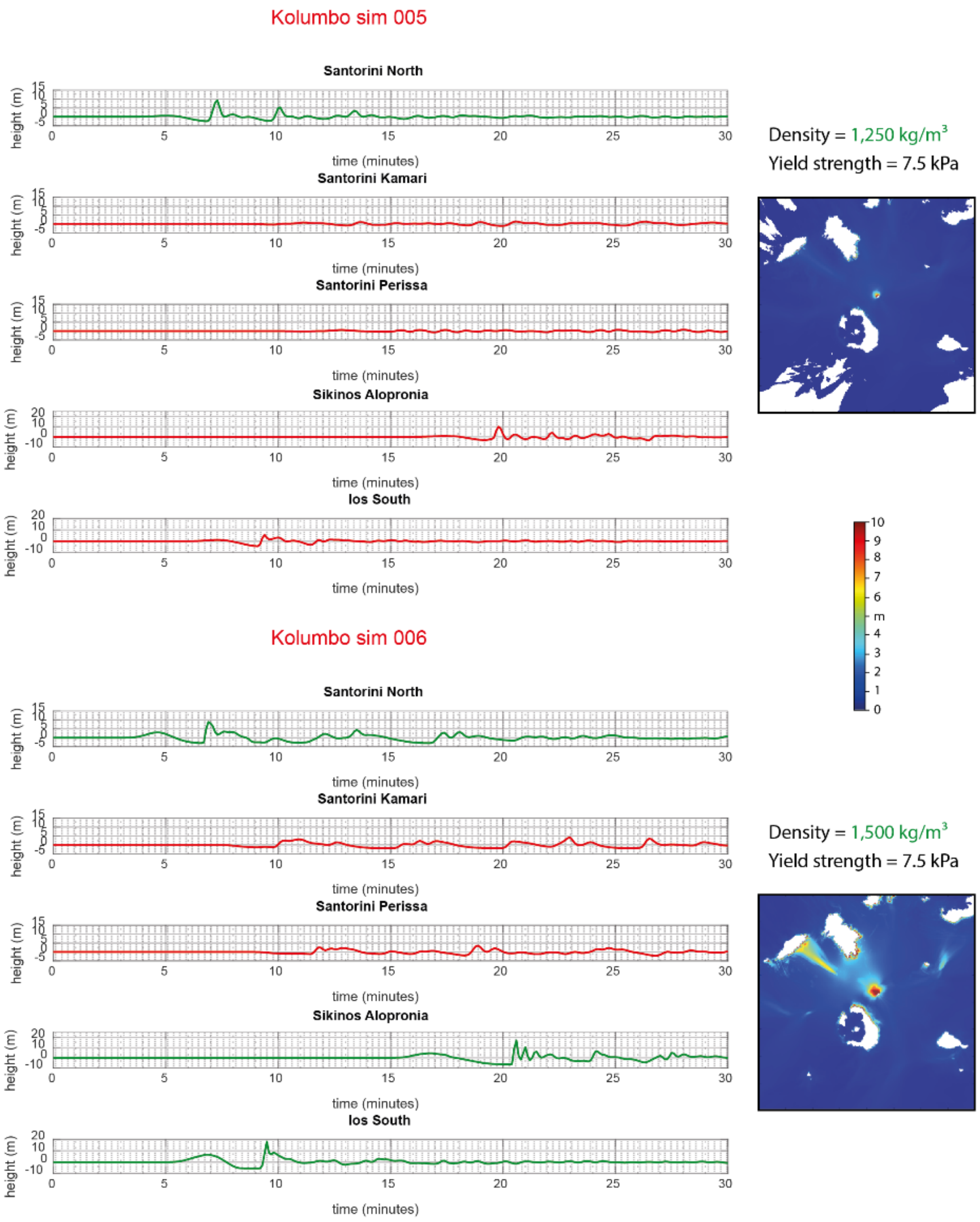

Figure S6 | Virtual tide gauges for simulations sim 005 and sim 006 (red: inconsistent with historical accounts; green: in agreement with historical accounts). Threshold tsunami heights from eyewitness accounts used to evaluate the simulation results: 7.5 m at northern Santorini, 5 m at Perissa, 5 m Kamari, 10 m at southern Ios and 10 m at Sikinos. A direct transfer of inundation observations to tsunami height for Sikinos is not possible and thus the Sikinos threshold is less reliable compared to the direct tsunami height information available for the other locations.

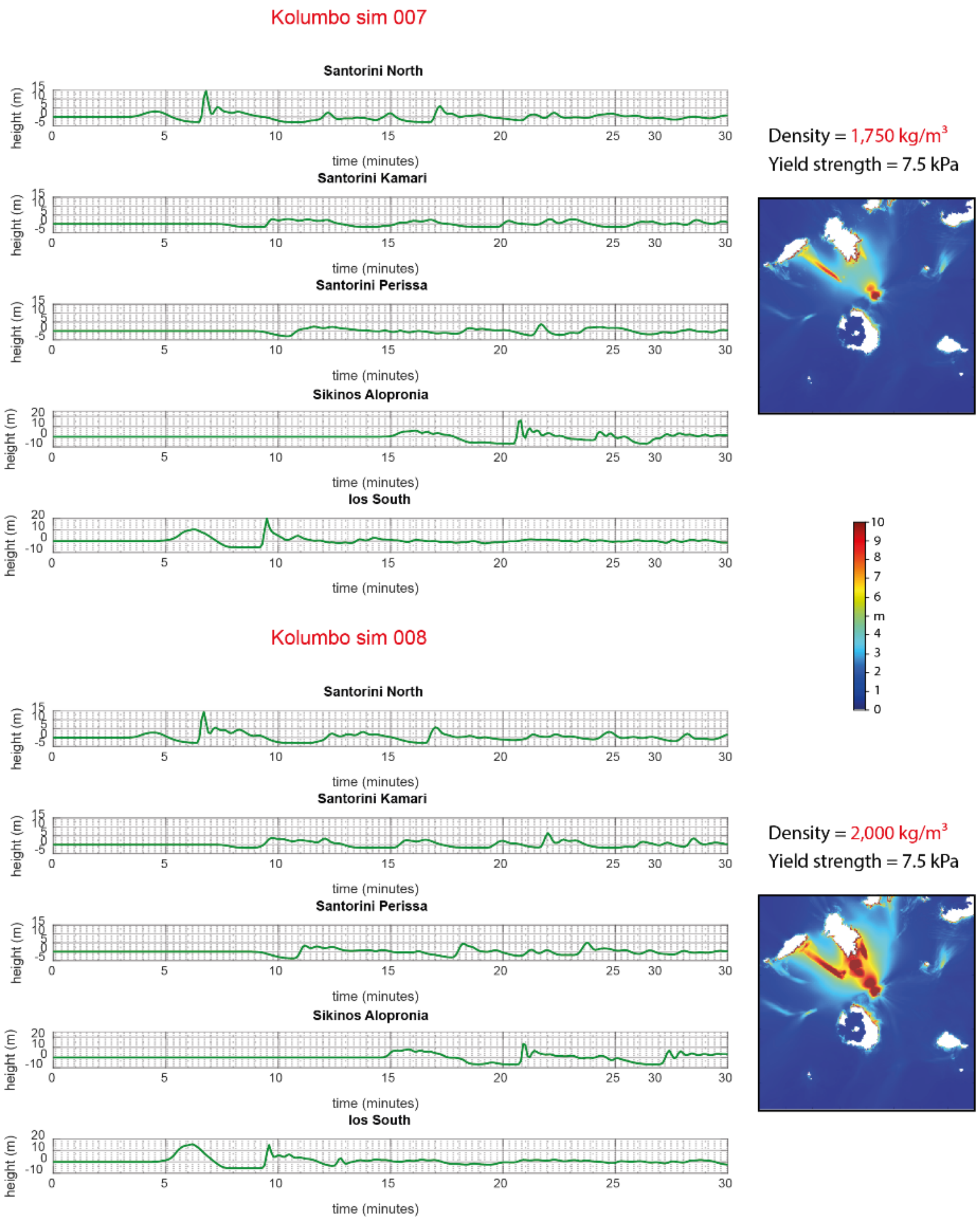

Figure S7 | Virtual tide gauges for simulations sim 007 and sim 008 (red: inconsistent with historical accounts; green: in agreement with historical accounts). Threshold tsunami heights from eyewitness accounts used to evaluate the simulation results: 7.5 m at northern Santorini, 5 m at Perissa, 5 m Kamari, 10 m at southern Ios and 10 m at Sikinos. A direct transfer of inundation observations to tsunami height for Sikinos is not possible and thus the Sikinos threshold is less reliable compared to the direct tsunami height information available for the other locations.

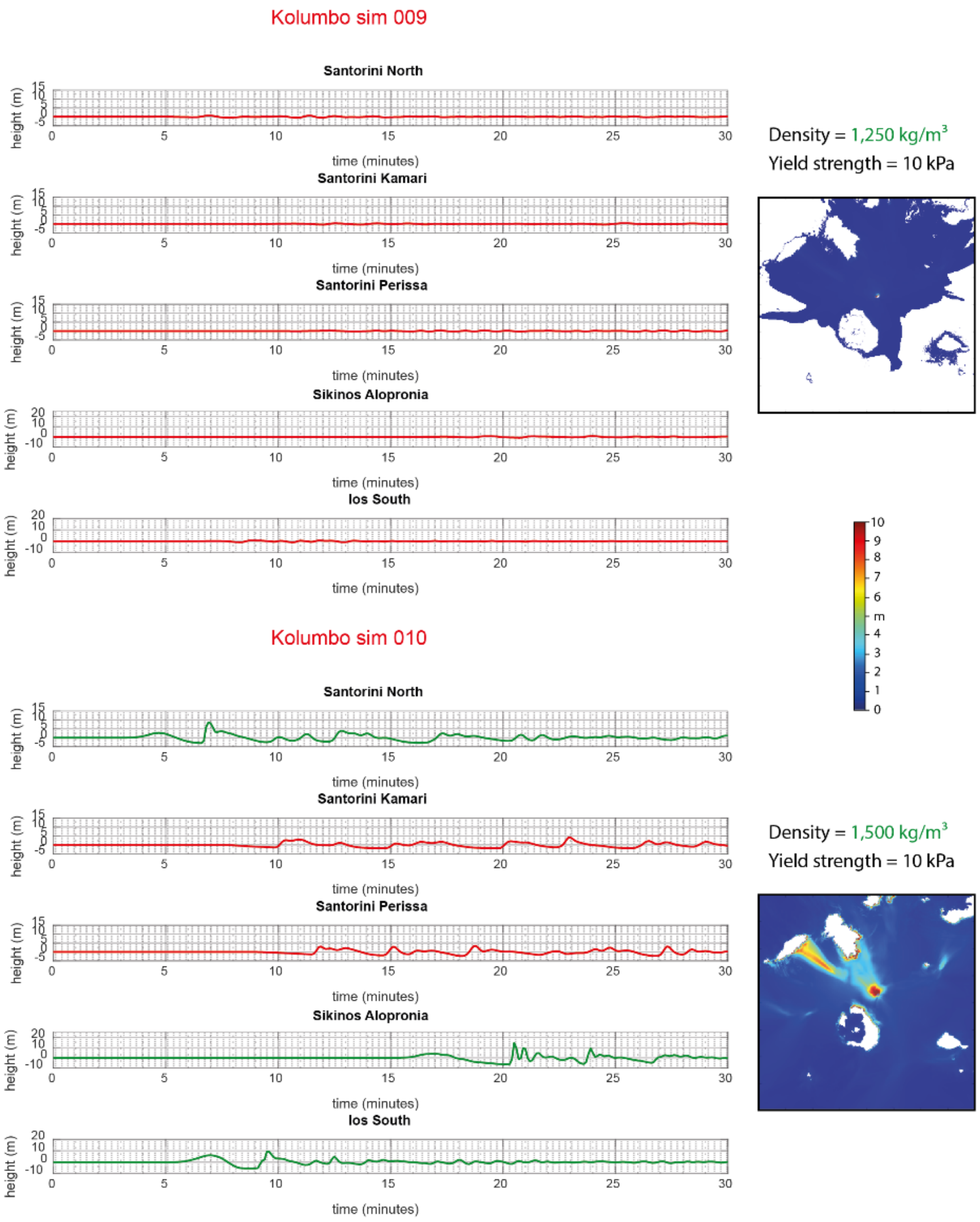

Figure S8 | Virtual tide gauges for simulations sim 009 and sim 010 (red: inconsistent with historical accounts; green: in agreement with historical accounts). Threshold tsunami heights from eyewitness accounts used to evaluate the simulation results: 7.5 m at northern Santorini, 5 m at Perissa, 5 m Kamari, 10 m at southern Ios and 10 m at Sikinos. A direct transfer of inundation observations to tsunami height for Sikinos is not possible and thus the Sikinos threshold is less reliable compared to the direct tsunami height information available for the other locations.

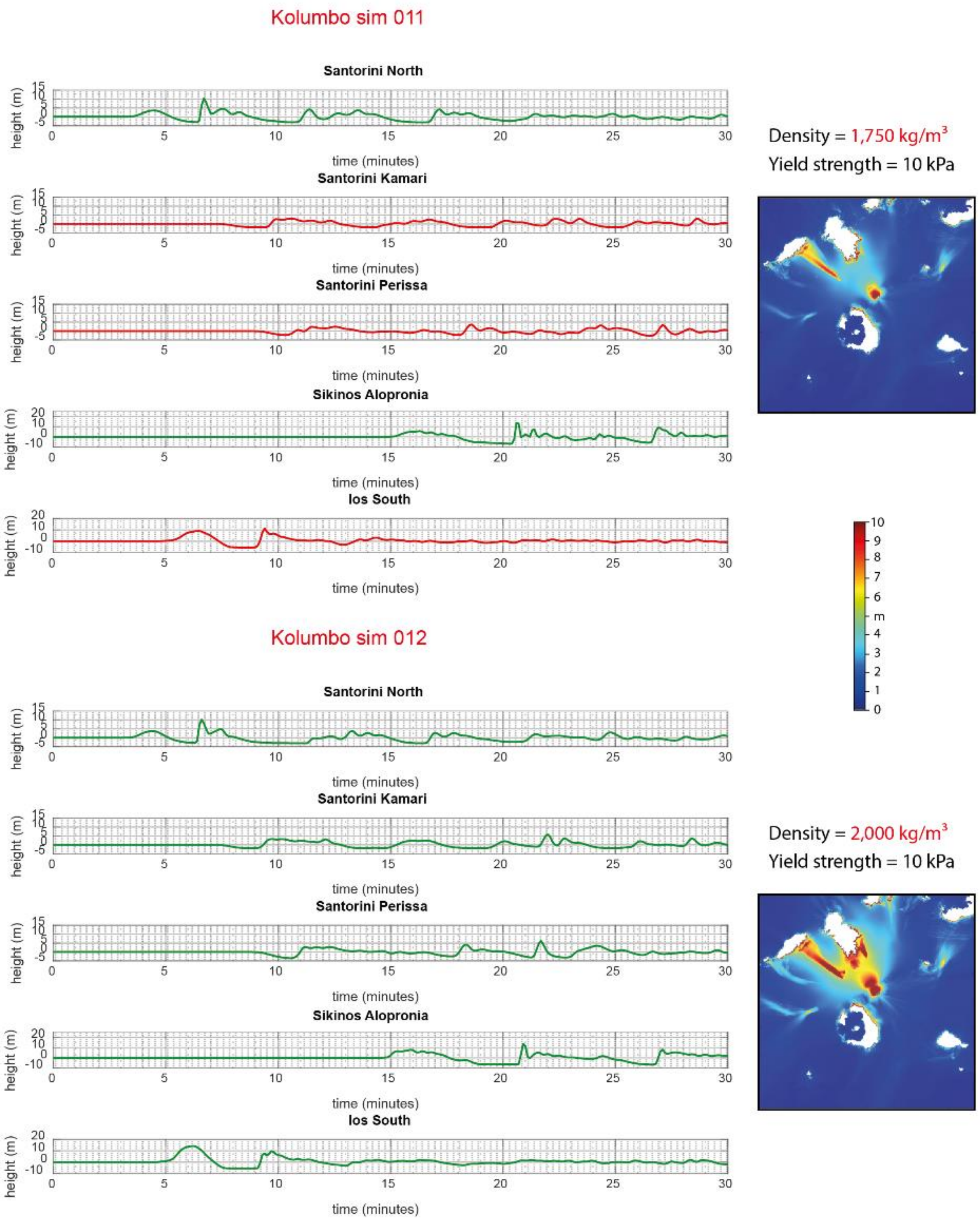

Figure S9 | Virtual tide gauges for simulations sim 011 and sim 012 (red: inconsistent with historical accounts; green: in agreement with historical accounts). Threshold tsunami heights from eyewitness accounts used to evaluate the simulation results: 7.5 m at northern Santorini, 5 m at Perissa, 5 m Kamari, 10 m at southern Ios and 10 m at Sikinos. A direct transfer of inundation observations to tsunami height for Sikinos is not possible and thus the Sikinos threshold is less reliable compared to the direct tsunami height information available for the other locations.

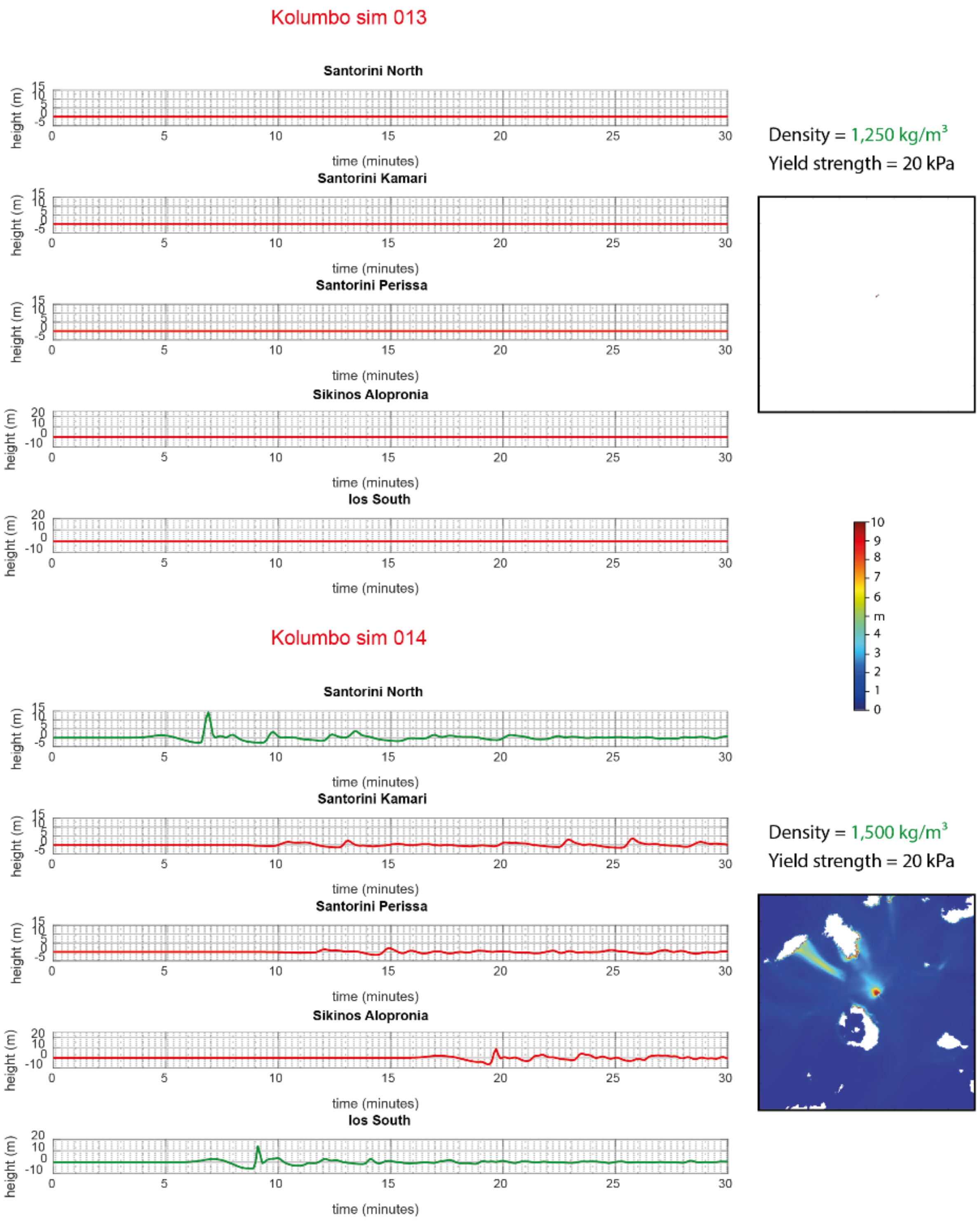

Figure S10 | Virtual tide gauges for simulations sim 013 and sim 014 (red: inconsistent with historical accounts; green: in agreement with historical accounts). Threshold tsunami heights from eyewitness accounts used to evaluate the simulation results: 7.5 m at northern Santorini, 5 m at Perissa, 5 m Kamari, 10 m at southern Ios and 10 m at Sikinos. A direct transfer of inundation observations to tsunami height for Sikinos is not possible and thus the Sikinos threshold is less reliable compared to the direct tsunami height information available for the other locations.

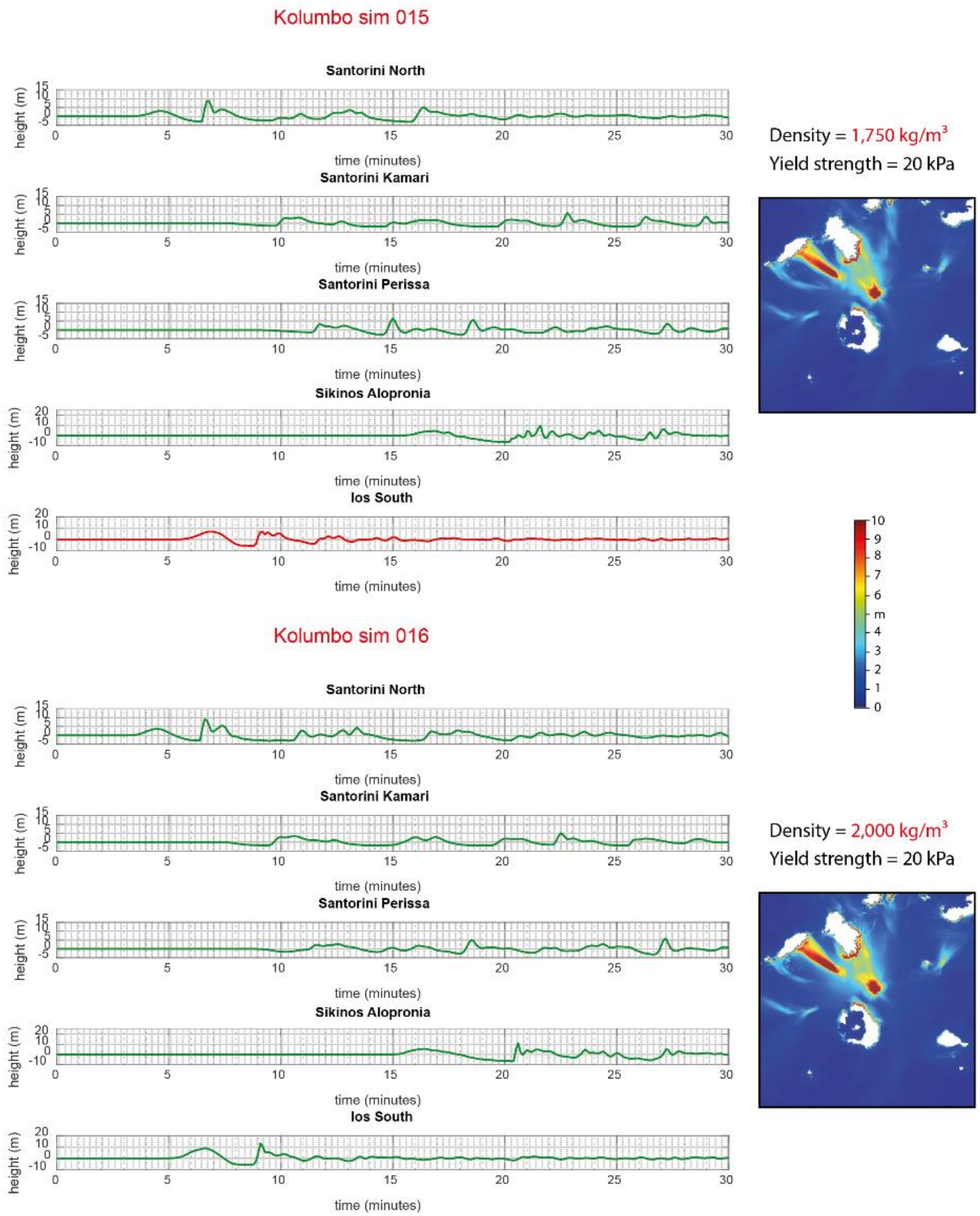

Figure S11 | Virtual tide gauges for simulations sim 015 and sim 016 (red: inconsistent with historical accounts; green: in agreement with historical accounts). Threshold tsunami heights from eyewitness accounts used to evaluate the simulation results: 7.5 m at northern Santorini, 5 m at Perissa, 5 m Kamari, 10 m at southern Ios and 10 m at Sikinos. A direct transfer of inundation observations to tsunami height for Sikinos is not possible and thus the Sikinos threshold is less reliable compared to the direct tsunami height information available for the other locations.

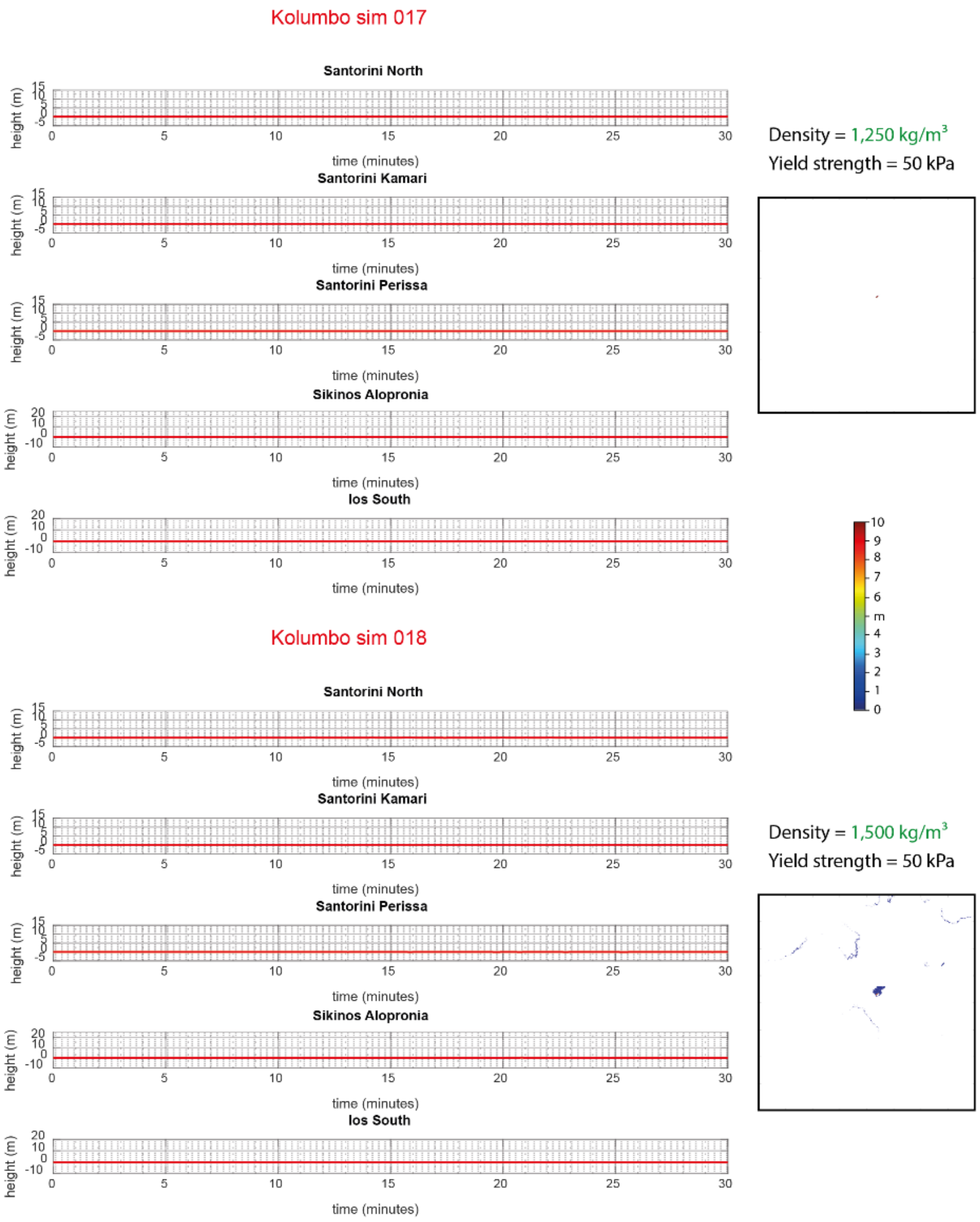

Figure S12 | Virtual tide gauges for simulations sim 017 and sim 018 (red: inconsistent with historical accounts; green: in agreement with historical accounts). Threshold tsunami heights from eyewitness accounts used to evaluate the simulation results: 7.5 m at northern Santorini, 5 m at Perissa, 5 m Kamari, 10 m at southern Ios and 10 m at Sikinos. A direct transfer of inundation observations to tsunami height for Sikinos is not possible and thus the Sikinos threshold hs is less reliable compared to the direct tsunami height information available for the other locations.

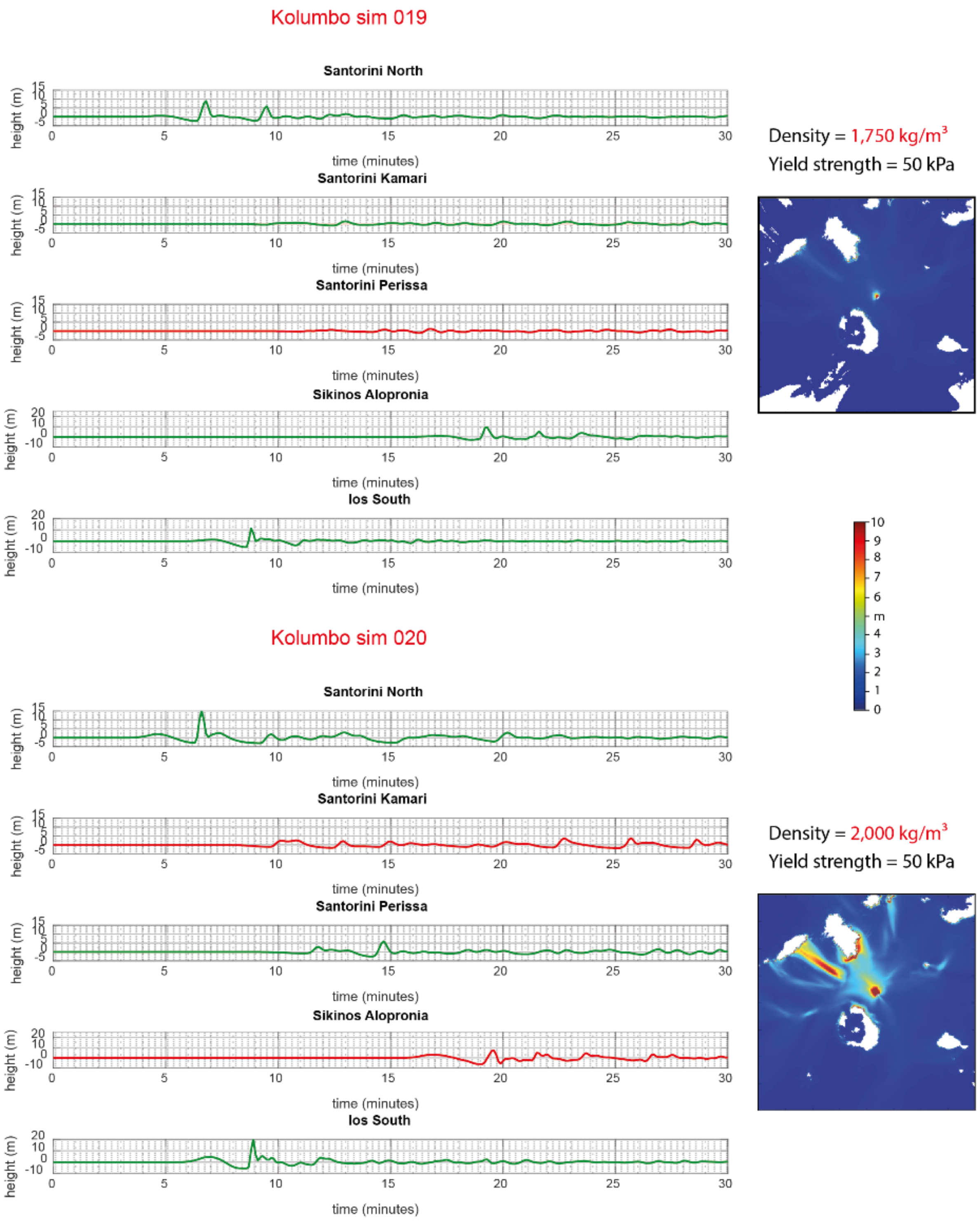

Figure S13 | Virtual tide gauges for simulations sim 019 and sim 020 (red: inconsistent with historical accounts; green: in agreement with historical accounts). Threshold tsunami heights from eyewitness accounts used to evaluate the simulation results: 7.5 m at northern Santorini, 5 m at Perissa, 5 m Kamari, 10 m at southern Ios and 10 m at Sikinos. A direct transfer of inundation observations to tsunami height for Sikinos is not possible and thus the Sikinos threshold is less reliable compared to the direct tsunami height information available for the other locations.

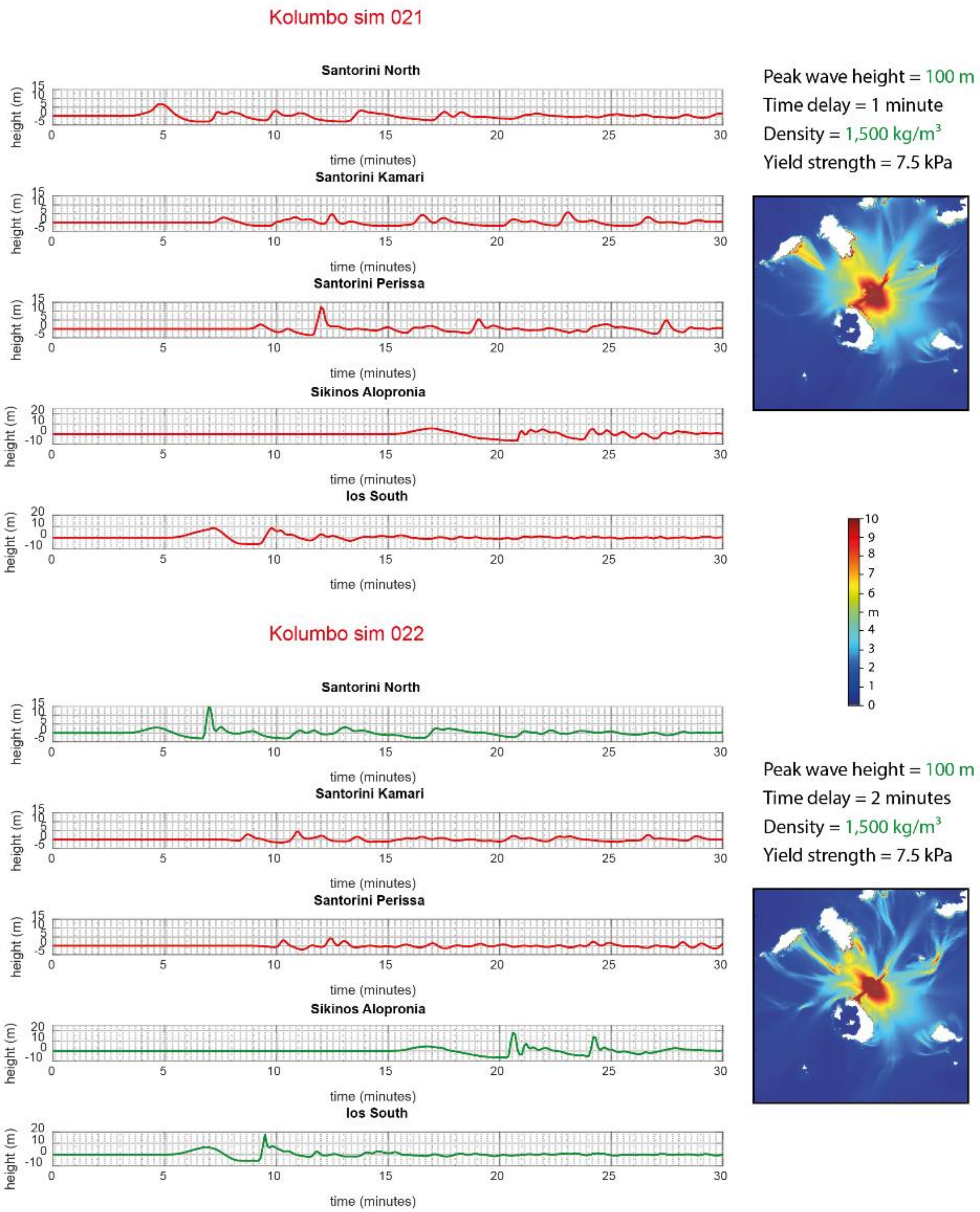

Figure S14 | Virtual tide gauges for simulations sim 021 and sim 022 (red: inconsistent with historical accounts; green: in agreement with historical accounts). Threshold tsunami heights from eyewitness accounts used to evaluate the simulation results: 7.5 m at northern Santorini, 5 m at Perissa, 5 m Kamari, 10 m at southern Ios and 10 m at Sikinos. A direct transfer of inundation observations to tsunami height for Sikinos is not possible and thus the Sikinos threshold is less reliable compared to the direct tsunami height information available for the other locations.

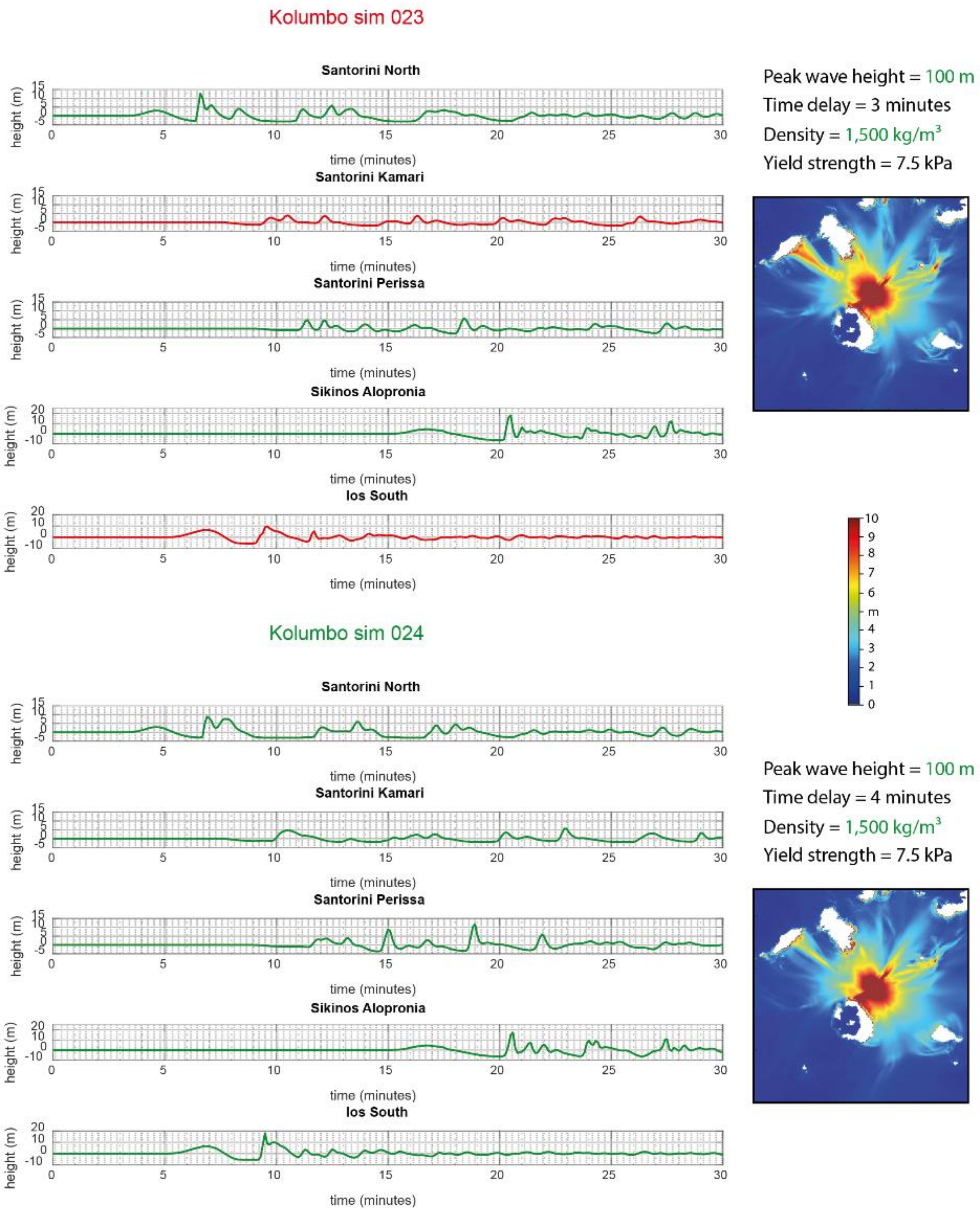

Figure S15 | Virtual tide gauges for simulations sim 023 and sim 024 (red: inconsistent with historical accounts; green: in agreement with historical accounts). Threshold tsunami heights from eyewitness accounts used to evaluate the simulation results: 7.5 m at northern Santorini, 5 m at Perissa, 5 m Kamari, 10 m at southern Ios and 10 m at Sikinos. A direct transfer of inundation observations to tsunami height for Sikinos is not possible and thus the Sikinos threshold is less reliable compared to the direct tsunami height information available for the other locations.

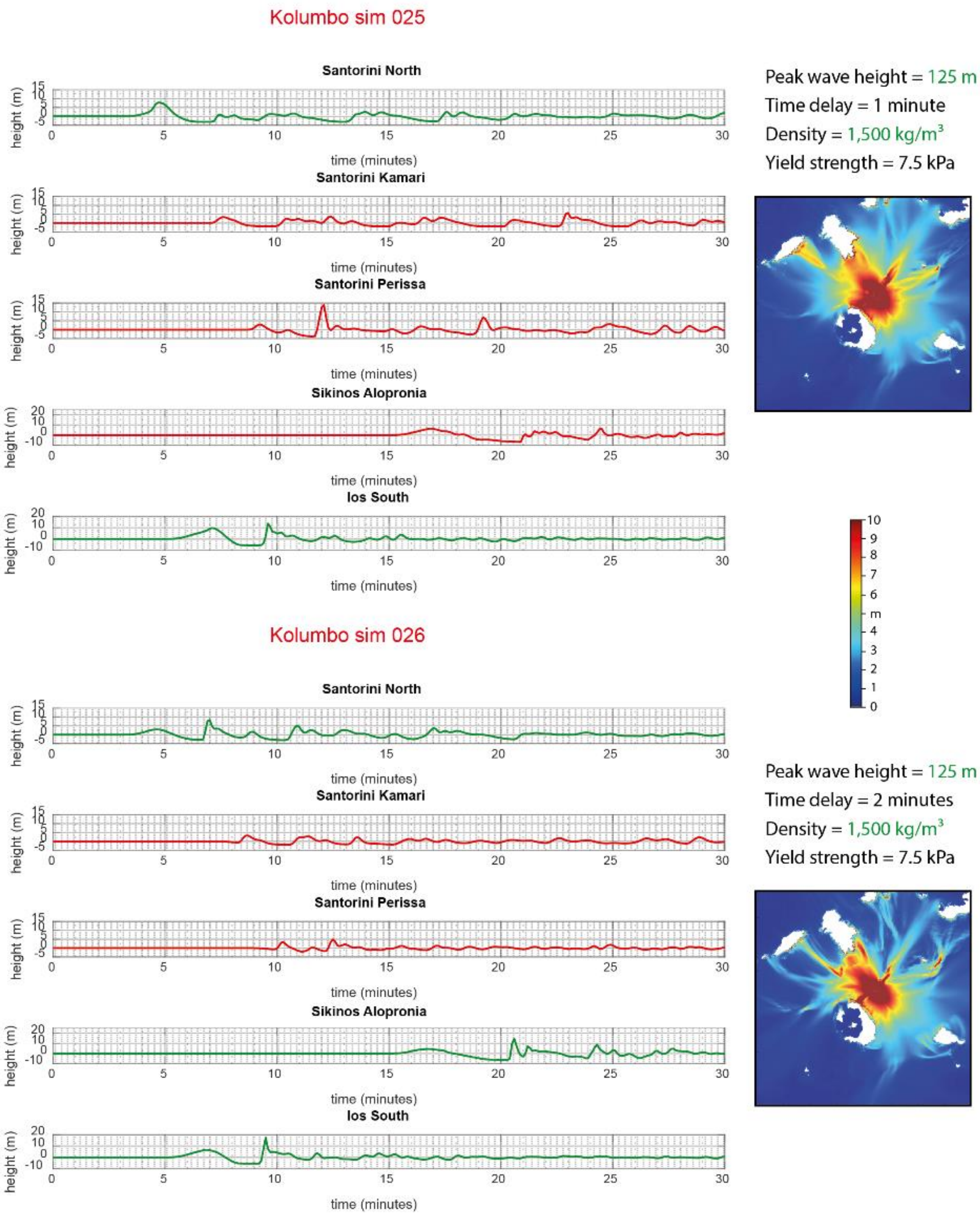

Figure S16 | Virtual tide gauges for simulations sim 025 and sim 026 (red: inconsistent with historical accounts; green: in agreement with historical accounts). Threshold tsunami heights from eyewitness accounts used to evaluate the simulation results: 7.5 m at northern Santorini, 5 m at Perissa, 5 m Kamari, 10 m at southern Ios and 10 m at Sikinos. A direct transfer of inundation observations to tsunami height for Sikinos is not possible and thus the Sikinos threshold is less reliable compared to the direct tsunami height information available for the other locations.

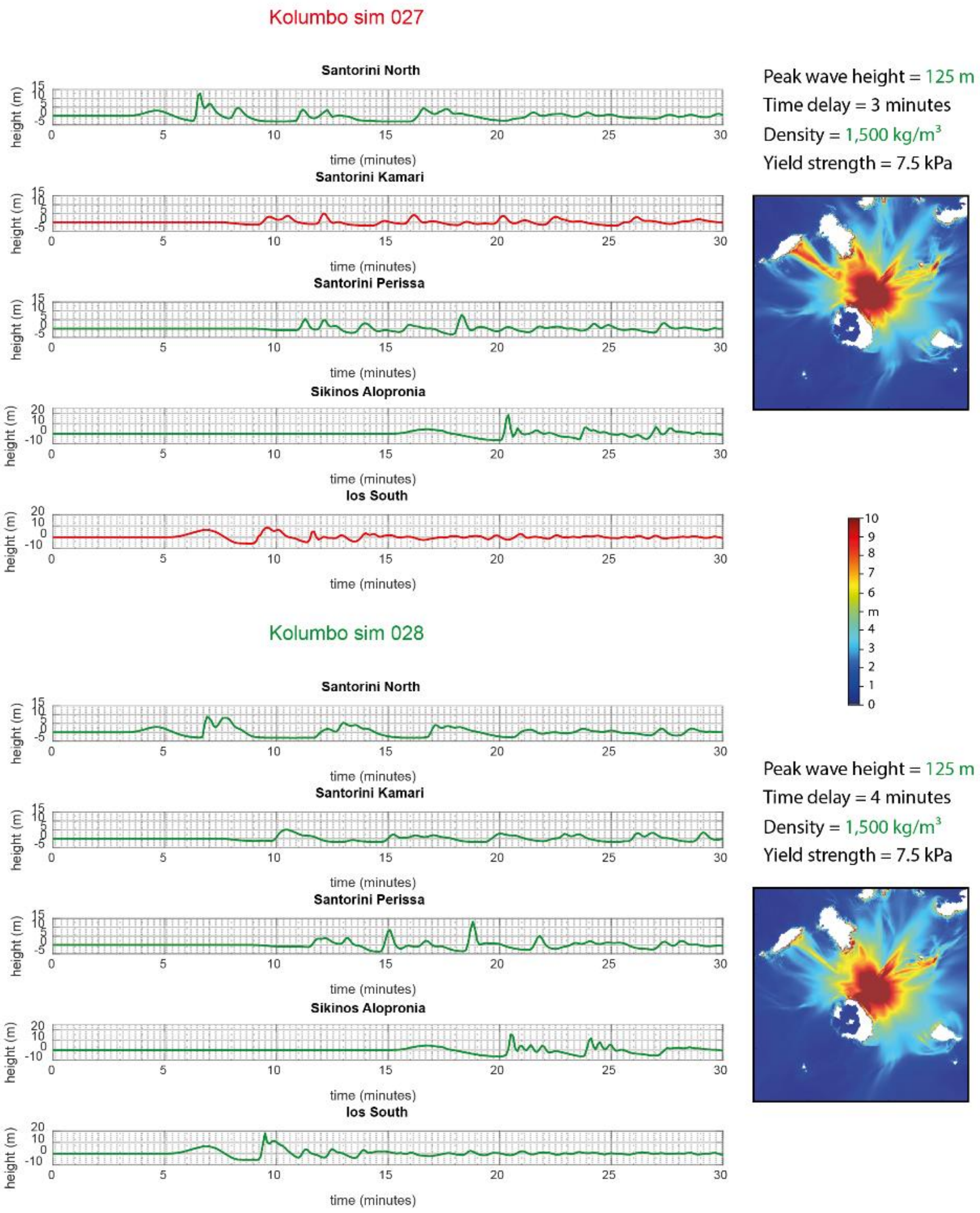

Figure S17 | Virtual tide gauges for simulations sim 027 and sim 028 (red: inconsistent with historical accounts; green: in agreement with historical accounts). Threshold tsunami heights from eyewitness accounts used to evaluate the simulation results: 7.5 m at northern Santorini, 5 m at Perissa, 5 m Kamari, 10 m at southern Ios and 10 m at Sikinos. A direct transfer of inundation observations to tsunami height for Sikinos is not possible and thus the Sikinos thresholdhas is less reliable compared to the direct tsunami height information available for the other locations.

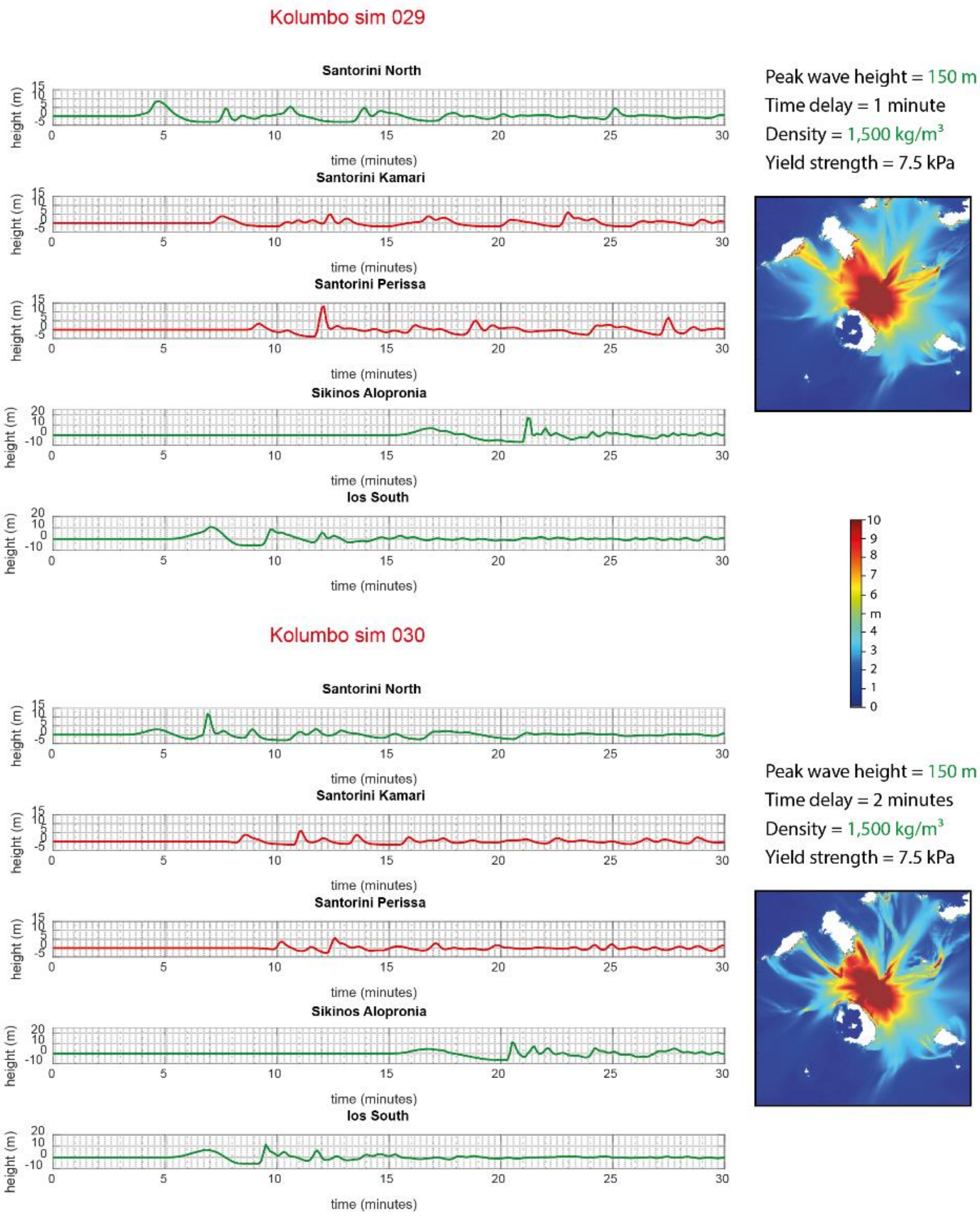

Figure S18 | Virtual tide gauges for simulations sim 029 and sim 030 (red: inconsistent with historical accounts; green: in agreement with historical accounts). Threshold tsunami heights from eyewitness accounts used to evaluate the simulation results: 7.5 m at northern Santorini, 5 m at Perissa, 5 m Kamari, 10 m at southern Ios and 10 m at Sikinos. A direct transfer of inundation observations to tsunami height for Sikinos is not possible and thus the Sikinos threshold is less reliable compared to the direct tsunami height information available for the other locations.

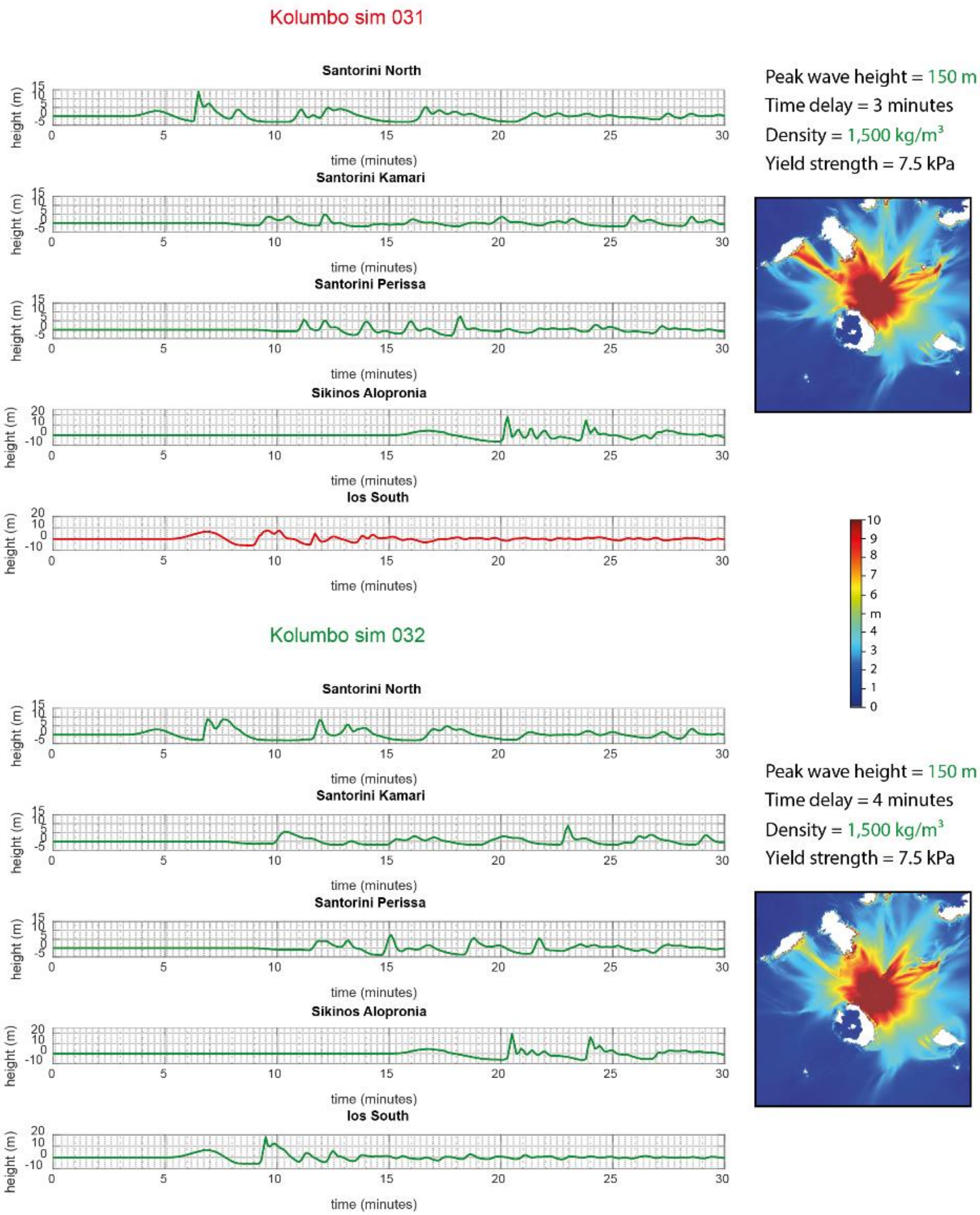

Figure S19 | Virtual tide gauges for simulations sim 031 and sim 032 (red: inconsistent with historical accounts; green: in agreement with historical accounts). Threshold tsunami heights from eyewitness accounts used to evaluate the simulation results: 7.5 m at northern Santorini, 5 m at Perissa, 5 m Kamari, 10 m at southern Ios and 10 m at Sikinos. A direct transfer of inundation observations to tsunami height for Sikinos is not possible and thus the Sikinos threshold is less reliable compared to the direct tsunami height information available for the other locations.

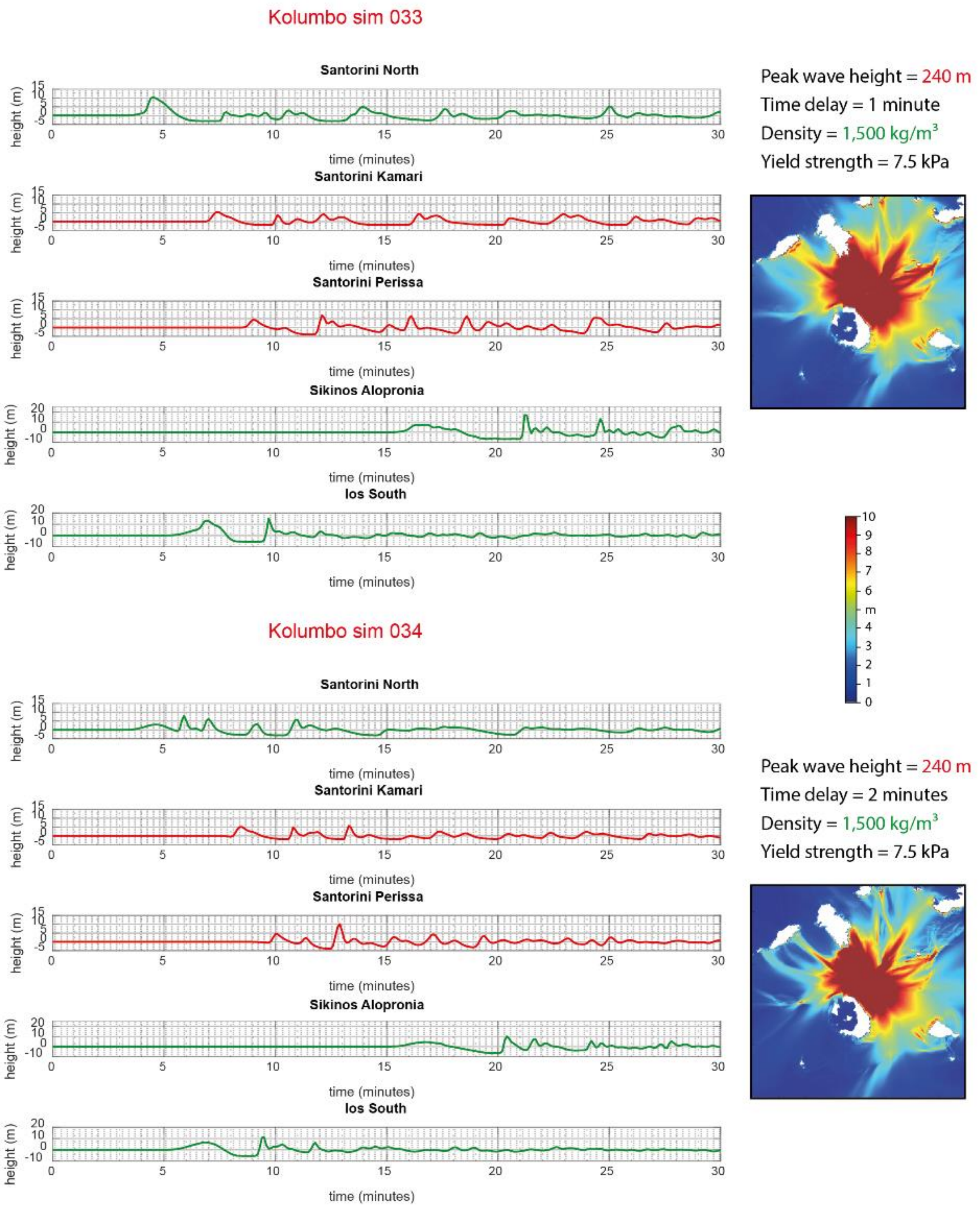

Figure S20 | Virtual tide gauges for simulations sim 033 and sim 034 (red: inconsistent with historical accounts; green: in agreement with historical accounts). Threshold tsunami heights from eyewitness accounts used to evaluate the simulation results: 7.5 m at northern Santorini, 5 m at Perissa, 5 m Kamari, 10 m at southern Ios and 10 m at Sikinos. A direct transfer of inundation observations to tsunami height for Sikinos is not possible and thus the Sikinos threshold is less reliable compared to the direct tsunami height information available for the other locations.

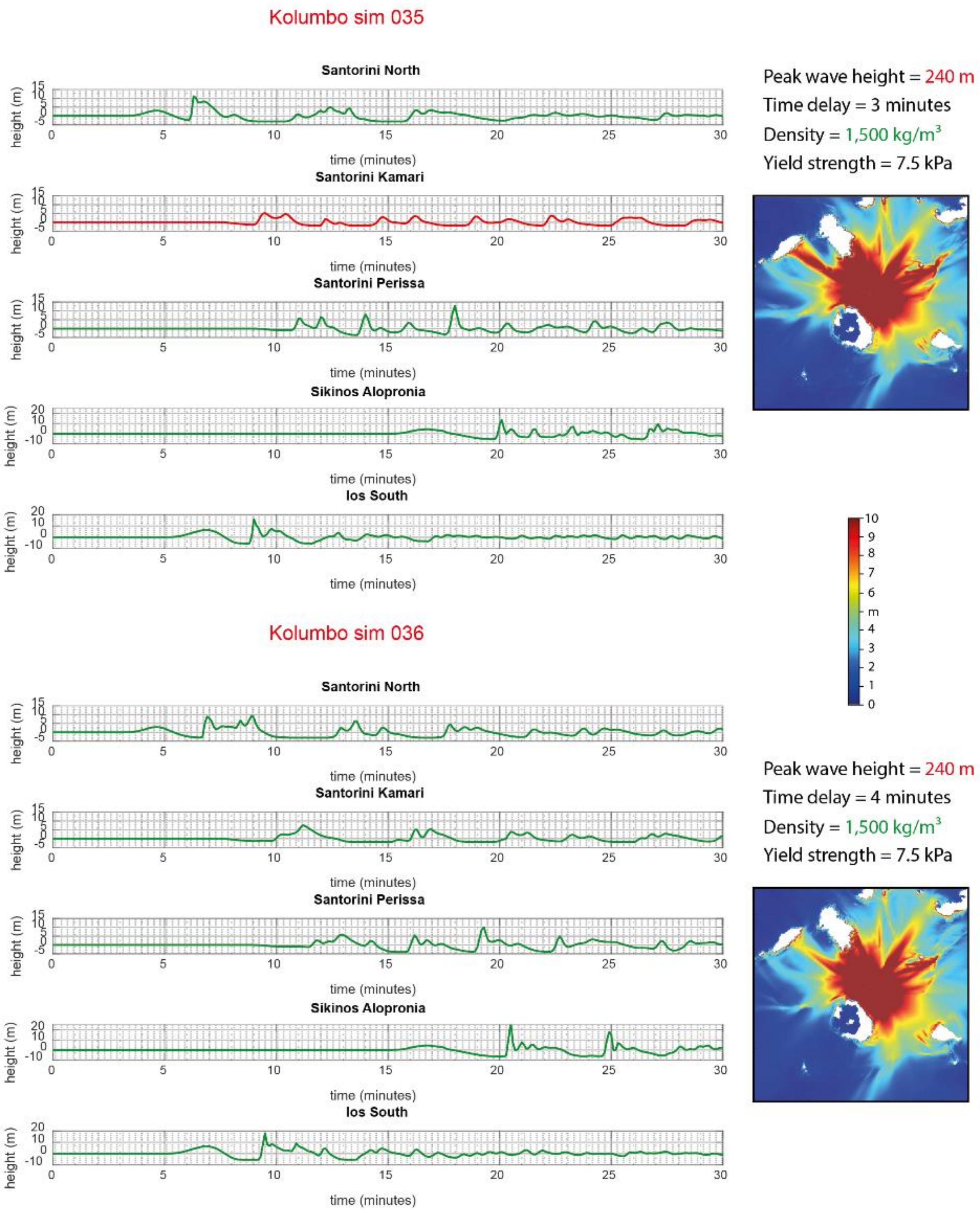

Figure S21 | Virtual tide gauges for simulations sim 035 and sim 036 (red: inconsistent with historical accounts; green: in agreement with historical accounts). Threshold tsunami heights from eyewitness accounts used to evaluate the simulation results: 7.5 m at northern Santorini, 5 m at Perissa, 5 m Kamari, 10 m at southern Ios and 10 m at Sikinos. A direct transfer of inundation observations to tsunami height for Sikinos is not possible and thus the Sikinos threshold is less reliable compared to the direct tsunami height information available for the other locations.

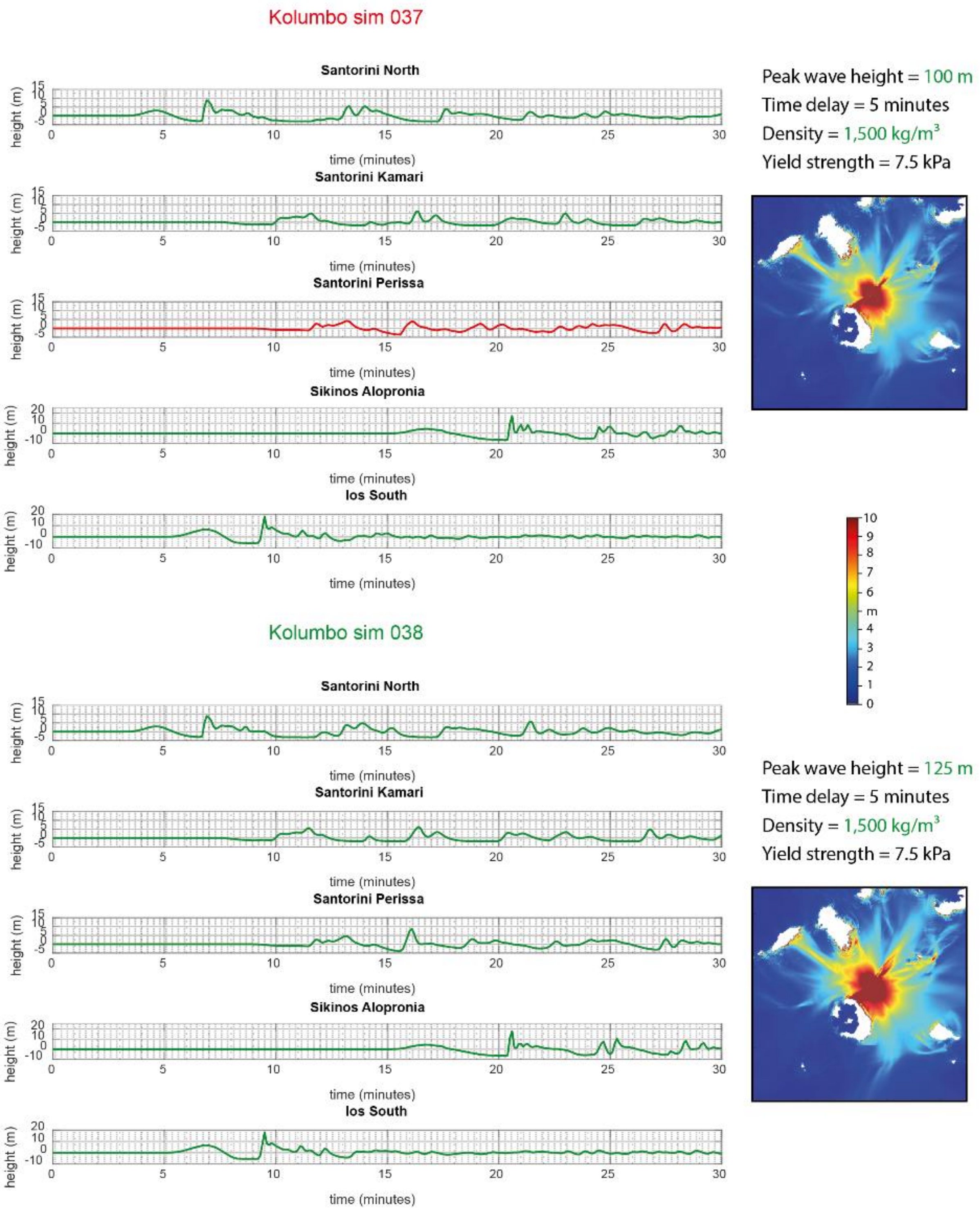

Figure S22 | Virtual tide gauges for simulations sim 037 and sim 038 (red: inconsistent with historical accounts; green: in agreement with historical accounts). Threshold tsunami heights from eyewitness accounts used to evaluate the simulation results: 7.5 m at northern Santorini, 5 m at Perissa, 5 m Kamari, 10 m at southern Ios and 10 m at Sikinos. A direct transfer of inundation observations to tsunami height for Sikinos is not possible and thus the Sikinos threshold is less reliable compared to the direct tsunami height information available for the other locations.

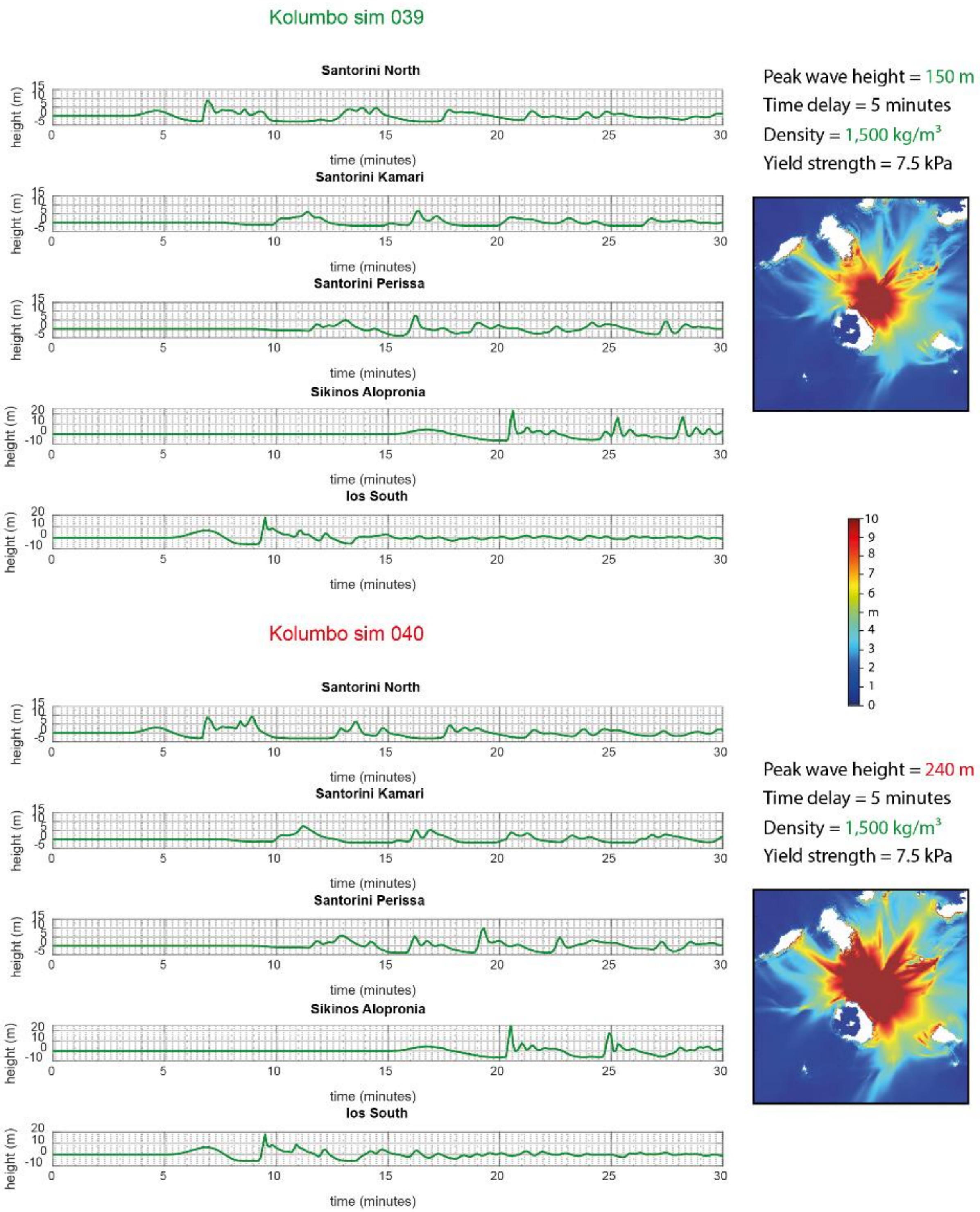

Figure S23 | Virtual tide gauges for simulations sim 039 and sim 040 (red: inconsistent with historical accounts; green: in agreement with historical accounts). Threshold tsunami heights from eyewitness accounts used to evaluate the simulation results: 7.5 m at northern Santorini, 5 m at Perissa, 5 m Kamari, 10 m at southern Ios and 10 m at Sikinos. A direct transfer of inundation observations to tsunami height for Sikinos is not possible and thus the Sikinos threshold is less reliable compared to the direct tsunami height information available for the other locations.

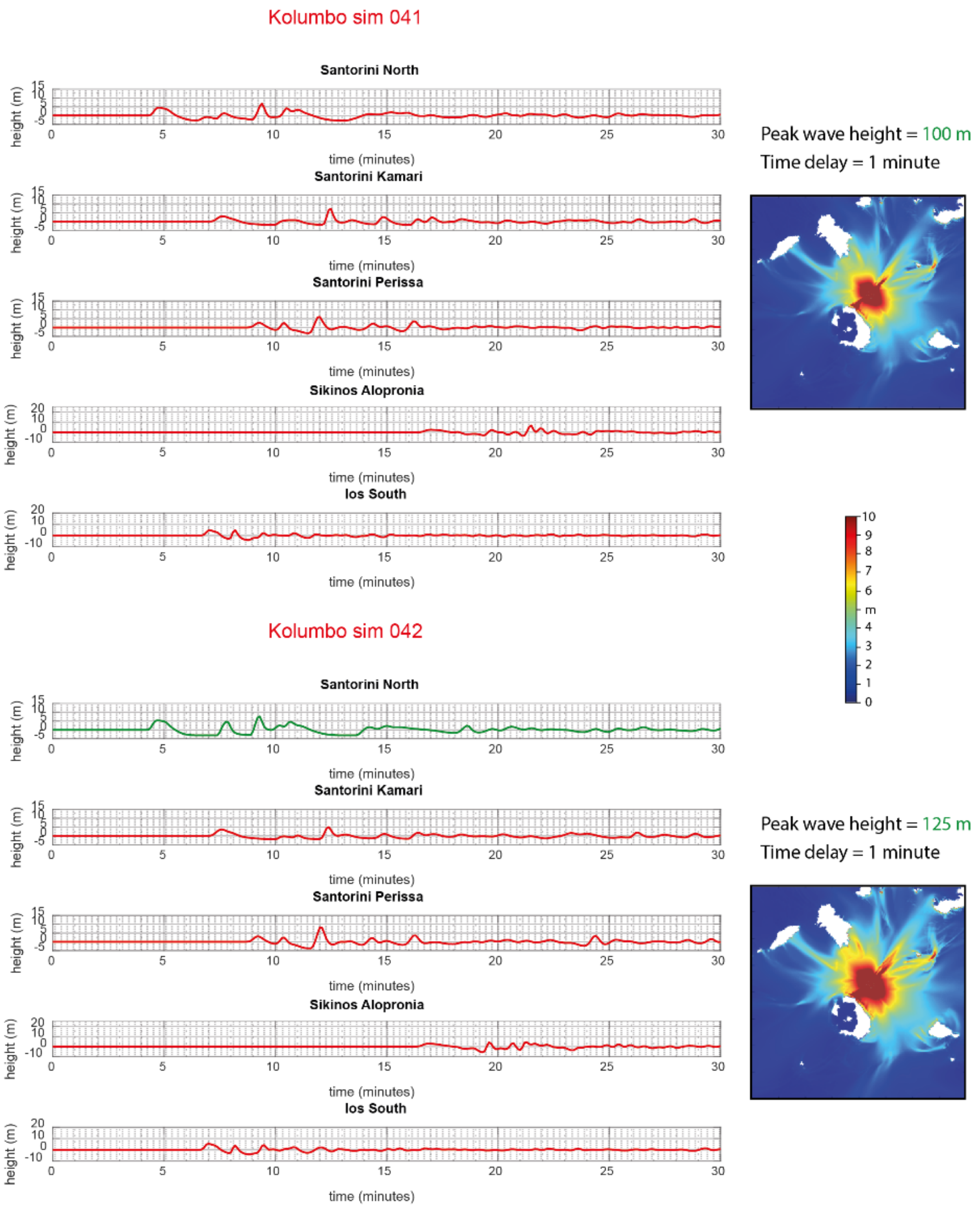

Figure S24 | Virtual tide gauges for simulations sim 041 and sim 042 (red: inconsistent with historical accounts; green: in agreement with historical accounts). Threshold tsunami heights from eyewitness accounts used to evaluate the simulation results: 7.5 m at northern Santorini, 5 m at Perissa, 5 m Kamari, 10 m at southern Ios and 10 m at Sikinos. A direct transfer of inundation observations to tsunami height for Sikinos is not possible and thus the Sikinos threshold is less reliable compared to the direct tsunami height information available for the other locations.

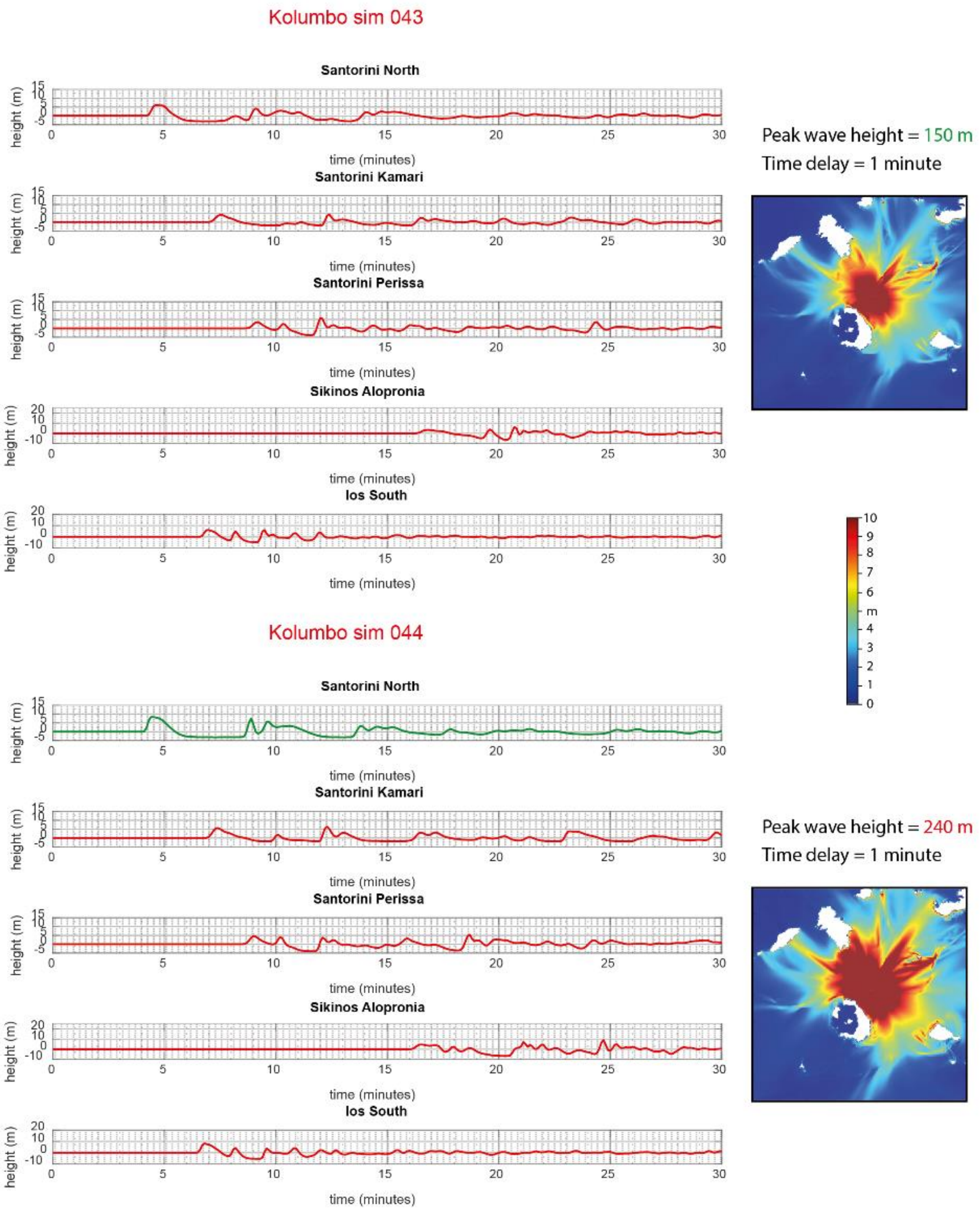

Figure S25 | Virtual tide gauges for simulations sim 043 and sim 044 (red: inconsistent with historical accounts; green: in agreement with historical accounts). Threshold tsunami heights from eyewitness accounts used to evaluate the simulation results: 7.5 m at northern Santorini, 5 m at Perissa, 5 m Kamari, 10 m at southern Ios and 10 m at Sikinos. A direct transfer of inundation observations to tsunami height for Sikinos is not possible and thus the Sikinos threshold is less reliable compared to the direct tsunami height information available for the other locations.
